# Complications During Maxillary Sinus Augmentation Associated with Interfering Septa: A New Classification of Septa

**DOI:** 10.2174/1874210601711010140

**Published:** 2017-03-22

**Authors:** Tassos Irinakis, Valentin Dabuleanu, Salwa Aldahlawi

**Affiliations:** 1 Faculty of Dentistry, University of British Columbia, Vancouver, BC, Canada; 2 Private practice, Dabuleanu Dental 2 Finch Avenue, West Toronto, ON, Canada; 3 Faculty of Dentistry, Umm alqura University, Mecca, Saudi Arabia

**Keywords:** Direct sinus lift, Maxillary septum classification, Schneiderian membrane perforation, Dental implant, Marginal bone loss, Post operative complication, Bone graft

## Abstract

**Purpose:**

A new classification of maxillary sinus interfering septa based on its orientation is presented along with its relationship to the prevalence and severity of sinus membrane perforations. Additionally, the impact of membrane perforation on post-operative complications and marginal bone loss during the first year of loading is evaluated.

**Materials & Methods:**

Retrospective chart review of 79 consecutive sinus lift procedures with lateral window technique and 107 implants. Preoperative Cone Beam Computed Tomography (CBCT) images were evaluated for the incidence and the direction of maxillary septa. Chart notes were examined for the incidence of membrane perforation and postoperative complications. Measurements of mesial and distal marginal bone levels and average bone resorption adjacent to each implant were calculated in intraoral radiographs taken at implant placement and during follow up appointments.

**Results:**

Interfering septa were identified in 48.1 percent of sinuses. 71.1 percent of them had the septum oriented in a buccal-lingual direction (Class I). The overall incidence of membrane perforation was 22.8 percent, and the presence of an interfering septum on CBCT scan was found to be significantly associated with the occurrence of a sinus membrane perforation (*P*<0.001). The mean implant marginal bone loss for sinuses, which did not experience a membrane perforation, was 0.6±0.8mm, compared with 0.9 ± 0.9 mm for the sinuses that did experience a perforation (*P* = 0.325).

**Conclusion:**

Septa should be identified, classified and managed with a meticulous attention to technical details. A classification based on the septal orientation is proposed since the orientation of the septa can complicate the surgical procedure and requires modification of the surgical technique.

## INTRODUCTION

The posterior maxilla represents a unique challenge when planning implant prosthetic rehabilitation of edentulous sites. Common problems facing the clinician are the lack of bone volume due to resorption of the alveolar process and pneumatization of the maxillary sinuses as well as poor bone quality. Sinus lift surgery is the most common procedure in use today in conjunction with implant placement in the posterior maxilla with reported similar outcomes of implant placed with or without sinus floor augmentation [1]. Implants placed in augmented sinuses had a survival rate range of 98.6% - 93.5% after 3-5 years of loading [2-4].

Schneiderian membrane perforation during sinus lift surgery has been reported to occur in 10-60% of all procedures [5-7]. It may lead to an increase in surgical challenges, time and postoperative complications. Careful preoperative treatment planning can reduce the risk of membrane perforation. Anatomical factors like the presence, location and direction of the maxillary septum, the thickness, and angles of maxillary sinus walls and the thickness of the Schneiderian membrane need to be carefully identified and examined in conventional and three-dimensional radiographic images. However, even careful preparation, reflection and mobilization of the membrane along anatomical irregularities, cannot always prevent perforation [8].

Underwood septa are osseous projections often present in the maxillary sinus and divide the sinus into smaller compartments. Septa’s main function is to act as masticatory force carrying struts during the dentate phase in life [9]. The term “interfering septa” is used to identify a septum lying directly above the alveolar ridge where sinus lift surgery was planned, and thus “interfered” during the preparation of the osseous window and/or the reflection of the Schneiderian membrane [10]. Overall, the prevalence of septa reported in the literature at the sinus level is between 16% and 48% [11-17].

Although a reduced implant survival following sinus membrane perforations has been reported, [5, 18] none of the studies have specifically investigated the relationship between the orientation of maxillary sinus septa and the occurrence of intra-surgical complications, such as sinus membrane perforation, as well as postoperative complications, such as the development of graft necrosis and persistent sinusitis. In addition, none of them evaluated long-term implant marginal bone loss following such complications. While a variety of techniques have been discussed with respect to the intra-surgical management of perforation [19-21], surgical abandonment has also been documented, particularly in cases of large perforations [6]. For these reasons, an increased understanding of the risks posed by interfering sinus septa is of high value to both the surgeon and the patient.

The primary objectives of the present study were to propose a classification system based on the orientation of sinus septa and to assess the prevalence and severity of sinus membrane perforation complications as they relate to the presence of sinus septa and this classification. Secondary objectives included the evaluation of the impact of membrane perforation on post-operative complications and marginal bone loss during the first year of loading as measured on intraoral radiographs.

## MATERIALS AND METHODS

Study design: This study is a retrospective chart review of consecutive patients who underwent lateral window sinus lift procedure. Sinus lift and implant surgeries were performed in partially dentate and edentulous patients, in the posterior maxillary arch, utilizing either a one- or two-stage sinus lift approach. Residual ridge height was 0.8-8 mm. All partially dentate patients had either been previously treated for periodontal disease and were receiving maintenance therapy, or did not show any signs or symptoms of periodontal disease. The inclusion criteria for this study were: Patients who had at least one direct sinus lift surgery performed by one experienced periodontist (T.I) and in patients who also received implant therapy, the availability of periapical radiographs representing implants with clearly discernible threads during placement as well as during follow-up appointments.

### Surgical Procedure

All procedures were carried under local anesthesia. The flap design included a mid crestal incision over the edentulous ridge and divergent vertical incisions to allow for a passive flap approximation at the end of the procedure. Osseous lateral window was created using a combination of rotary burs and Piezo electrical tips. An oval shaped – rounded corners window of adequate size and width to allow appropriate use of sinus elevation instrument was created. The detecting of interfering septa in the preoperative CTCB required the modification of the window to resemble a W or a kidney. Once access to the sinus has been gained, a combination of Piezo electrical tips and hand instruments was used for the careful reflection of the Schneiderian membrane. Identification of a sinus septum required a careful and gentle elevation of the membrane around and off the bony septum. Membrane integrity was assessed visually and by asking the patients to blow their nose gently to detect the free movement of the membrane. If a perforation was detected, it was classified based on size into small (<5 mm) or large (≥5 mm). The perforation was repaired using an internally placed membrane (NeoMem by Citagenix Inc, Canada) following a parachute technique [10]. One of the four bone grafts was compacted into the sinus. Dental implants were placed simultaneously only in cases where implant stability could be achieved (> 3 mm of initial alveolar bone height). Otherwise, implant placement was delayed and done at a separate procedure. Resorbable membrane was used to cover the lateral window (Bio-Gide by Geistlich, Switzerland). Flap edges were approximated and a passive primary closure of the area was achieved. All patients received postoperative antibiotic (Amoxicillin 500 mg TID for 7 days or Clindamycin 300 mg TID for 7 days).

Patient follow-up: All patients were seen initially 7-10 days postoperatively and then a monthly follow-up visit was conducted. Delayed implant placement was done 6-9 months after the sinus augmentation and patients were followed up monthly during the healing period. At the integration check, osseointegration was verified radiographically, and implant stability was checked by resonance frequency analysis (Osstell ISQ by Osstell, Sweden) or a manual reverse torque test (<20 Ncm torque applied in a counterclockwise direction) when Ossstell was not available. Patients were then referred to the restorative dentist for the final prosthetic rehabilitation and were placed on an annual recall program.

### Data Collection

Patient charts were audited for patient’s demographic data, smoking and medical history. Significant medical history included: type I and type II diabetes mellitus, osteoporosis on bisphosphonate medication and sinusitis. Intra-operative notes documenting the steps of the surgical procedure and the presence of interfering septa, the occurrence of sinus membrane perforation, the size of the perforation, the type of bone graft used and the timing of implant placement and the type of implant used were reviewed. Post-operative notes documenting the healing period, and the occurrence of any postoperative complication like bleeding, infection including infected grafts, the development of persistent sinusitis for more than two months postoperatively, altered sensation, and soft tissue numbness or pain were analyzed.

Preoperative Cone Beam Computed Tomography (CBCT) images were evaluated using Kodak 3D Imaging v 2.4 software for the presence and the orientation of interfering septa. Interfering septa are defined as osseous projections located directly above the alveolar ridge where sinus lift surgery was planned and extended more than 2mm.

Intraoral radiographs taken at implant placement and during follow-up appointments were scanned and measured using Image J v1.46 software (National Institutes of Health, Maryland, USA). The coronal margin of the implant collar as well as the most coronal aspect of the bone-to-implant contact were used as reference points for linear measurements of marginal bone loss.

 Measurements of mesial and distal marginal bone levels adjacent to each implant were performed according to Piao, *et al.* [22]. The amount of bone resorption, which is the difference between the initial bone level and the bone level at the latest follow-up examination, was calculated and mesial and distal bone loss was determined for each implant. Thresholds of 1.5 mm and 1.0 mm of averaged marginal bone loss were selected for comparisons during the latest follow-up.

One examiner who was not involved in the treatment of patients evaluated all intraoral radiographs. Intra-examiner reliability was assessed in a sample of 25 randomly selected, and recordings of marginal bone loss at the latest follow-up were measured twice, once at baseline and after 3months. These duplicate recordings were highly correlated, with Pearson correlation coefficients of 0.988 (*P*<0.001) and 0.993 (*P*<0.001) at the mesial and distal measurements, respectively.

Descriptive statistics were used for all evaluated parameters. Patient and sinus characteristics were summarized in terms of frequencies and percentages for all variables assessed. This includes, at patient level: Age, gender, medical & smoking history, and at sinus level: septal class, size of perforation, type of bone graft used and if implants were placed simultaneously or not.

 The independent samples t-test and Fisher’s exact test were used to assess relationships between patient, bone grafting, implant placement timing, and intra-surgical related factors and outcome variables including marginal bone loss, the occurrence of sinus membrane perforation, and postoperative complications. The *P*-value of <0.05 was considered to be statistically significant.

## RESULTS

There were 79 direct sinus lifts done in 67 patients (62.7% females and 37.3% males) aged 55 ± 12.6 years. Only eight patients were current smokers. Twelve patients received bilateral sinus lift.

Simultaneous sinus lift and dental implant placement were performed in 31 sinuses, while delayed implant placement was performed in 48 sinuses. A total of 107 implants were placed (Table **1**). Of the implants placed, eleven were Nobel Replace Straight Groovy, forty-seven were Nobel Replace Tapered Groovy, one was Nobel Replace Select Straight, seven were Nobel Replace Select Tapered, six were Nobel Replace Conical Connection, six were Nobel Active, nine were Straumann Bone Level Sand-blasted, Large grit, Acid-etched (SLA), eighteen were Astra OsseoSpeed TX Straight, and two MIS SEVEN implants. One patient did not return for the completion of treatment following implant placement. Therefore, only 105 implants received an integration check. For one-stage implants, the integration check was done at a pre-determined time point after implant placement. For two-stage implants, the integration check was done at the time of the second stage surgery, and osseointegration was verified radiographically and by either an Osstell ISQ (n=73) or a manual reverse torque test (n=32). All implants that received an integration check had successfully osseointegrated, resulting in an overall survival rate of 100%. Average implant follow-up period after integration check was 12.8 months (1-37 m).

### Incidence of Septum

**Fig. (1):**
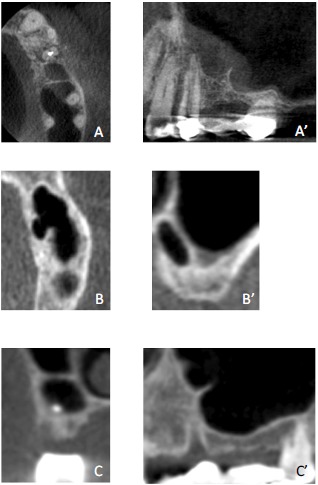
CBCT scan showing septum classification according to direction.A & A’: Class I septum in buccal lingual direction, A: Transverse Plane. A’: Sagittal PlaneB &B’: Class II septum in mesial distal direction, B: Transverse Plane. B’: Coronal PlaneC & C’: Class III septum in horizontal direction, C: Coronal Plan. C’: Sagittal Plane.

An evaluation of preoperative CBCT images revealed that interfering septa were recognized in 48.1% of sinuses. Interfering Septa were further classified according to their direction into Class I: septum oriented in a buccal-lingual direction (medial-lateral sinus direction or Coronal Plane). Class II: septum oriented in a mesial-distal direction (anterior-posterior sinus direction or Sagittal Plane). Class III: septum in a horizontal (shelf-like; Transverse Plane) orientation (off one of the medial or lateral walls). Class IV: septum of a combination of Class I, II, or III. (Fig. **1**).

The most common septal orientation identified was Class I septum in 34.2% (n=27) sinuses, the second most common were Class II and Class IV in 5.1% (n=4) sinuses. The least common was Class III, which was identified in 3.8% (n=3) sinuses. The incidence of different classes of septa is shown in Table **2**. It is important to notice that of all sinuses with septa identified on CBCT, 71.1% of them had class I septum.

### Intra-Operative Perforation

The overall occurrence of sinus membrane perforation was 22.8%, regardless of whether or not an interfering septum was visualized on the pre-operative CBCT image. The incidence of Small (<5 mm) and large (≥5 mm) membrane perforations were 12.7% (n=10) and 10.1% (n=8), respectively of all sinus lift procedures.

In sinuses where an interfering septum was visualized radiographically, the incidence of sinus membrane perforation was 44.7% and small and large perforation occurred in 26.3% and 18.4% of the cases, respectively. In sinuses where an interfering septum was not visualized radiographically, only one perforation occurred, leading to an incidence of 2.4%. The distribution of all perforations, both small and large, according to septal class is presented in Table **2**. An outline of clinical consideration is presented in Table **3**.

Due to the relatively small occurrence of radiographic Class II, III, and IV septum, a separate analysis of septal classes and the occurrence of sinus membrane perforations was not possible. However, the presence of an interfering radiographic septum on the pre-operative CBCT scan was found to be significantly associated with the occurrence of a sinus membrane perforation (*P* < 0.001) (Table **2**).

### Post-operative Complications

Post-operative complications occurred in only eight sinuses out of the 79 sinus treated, resulting in an occurrence rate of 10%. The majority of the complications relate to postoperative graft infection (5%). The incidence of other complications was 1.3%. Infection was treated with appropriate antibiotic.

Only two cases had intra-surgical membrane perforation followed by other complications; therefore, intra-surgical membrane perforation was not associated with the occurrence of postoperative complications (*P*=1.000). One patient had severe post-operative pain that radiates to the ipsilateral ear and was managed by pain medication. Another patient complained of facial asymmetry at the side of the procedure. However, facial symmetry improved as the soft tissue healing progressed. None of the complications resulted in the failure of the sinus augmentation.

There were four types of bone graft material used in sinus lifts: injectable paste allograft (n=26), particulate allograft (n=25), BioOss particulate xenograft (n=9), and particulate alloplast (n=19). When comparing the type of graft material used for the incidence of complications, it was found that injectable paste graft was significantly associated with a higher incidence of complications compared to particulate grafts (*P*=0.014).

### Patient and Procedure’s Factors Related to Membrane Perforation and Post-operative Complications

The study population was divided into three age groups: those younger than 45 years (n=12), those aged 45-64 (n=37), and those aged 65 years or older (n=18).

The incidence of membrane perforation among different age groups is presented in Table **1**. Although the oldest age group experienced the highest proportion of small and large perforations, this difference was not statistically significant (*P*=0.422). The youngest age group experienced the smallest proportion of other complications and the difference was only minimal and not statistically significant (*P*=1.000).

Female patients showed a larger proportion of major complications; however, the difference was not statistically significant (p=0.700). No gender difference was discovered with respect to the occurrence of membrane perforations.

Smokers had a higher occurrence of membrane perforations and nonsmokers had a higher occurrence of postoperative complications. However, the difference was not statistically significant (*P*=0.672) and (*P*=0.582), respectively. The presence of one significant medical history (type I and type II diabetes mellitus, osteoporosis on bisphosphonate medication and sinusitis) was not associated with the occurrence of membrane perforation or postoperative complications (*P*=0.760) and (*P*=0.424) respectively.

Out of the 79 sinuses evaluated in this study, 48 sinuses did not have sufficient bone for immediate implant placement and were augmented in a staged approach. The membrane perforation and postoperative complication rates in this group were 18.8% and 10.4%, respectively. Simultaneous implant placement with the sinus lift was found to be associated with an increased proportion of sinus membrane perforations when compared to staged approach, although the results were not statistically significant (*P*=0.410). Both groups were, in fact, found to be associated with equal proportions of postoperative complications, 9.7% and 10.4%, respectively (*P*=1.000) (Table **1**). 

### Implant Marginal Bone Loss

**Fig. (2):**
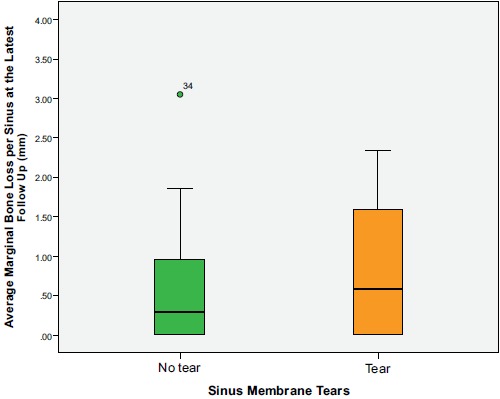
Average implant marginal bone loss according to sinus membrane perforation. Independent Samples T-Test, *P* = 0.325, N = 37. The outlier corresponds to a patient with a non-contributory medical history.

The mean implant marginal bone loss for sinuses that did not experience a membrane perforation was 0.6±0.8 mm, compared with 0.9 ± 0.9 mm for sinuses that did experience a perforation (*P*=0.325) (Fig. **2**).

At both the 1.5 mm and 1.0 mm thresholds, the occurrence of a sinus membrane perforation was associated with an increased occurrence of marginal bone loss. However, neither result was statistically significant (*P*=0.410, *P*=0.215 respectively) (Table **4**).

## DISCUSSION

The prevalence of interfering septa at the sinus level within this study was 48.1%. As previously discussed, the overall prevalence of septa reported in the literature at the sinus level is between 16% and 48% [17, 23, 24] with a higher occurrence in edentulous subjects as compared to dentulous subjects [24]. The study confirms that careful preoperative evaluation of a three dimensional radiograph is important in preventing membrane perforation during the sinus lift procedure. Membrane perforation occurred only in 17 sinuses of the 38 sinuses in which septa were identified preoperatively. However, the presence of an interfering radiographic septum on the preoperative CBCT scan was significantly associated with the occurrence of a sinus membrane perforation. The overall membrane perforation in relation to the presence of septa was 44.7% in this study and is in agreement with previously reported data [18, 25, 26]. Of these studies, only Malkinson and Irinakis [10] retrospectively assessed the presence of interfering septa on preoperative CBCT scans of 52 direct sinus lift procedures. A statistically significant association between the presence of interfering septa and membrane perforations was not found at that time [10]. However, in their study, a high percentage of membrane perforation occurred in sinuses that did not have interfering septa (10.8%) as compared to the sinuses that had septa (13%). Schwarz, *et al.* [26] identified the presence of sinus septa and residual ridge height of <3.5 mm as main risk factors for membrane perforation in sinus lift surgery.

Identification and management of interfering septa are essential to predict and avoid complications during sinus augmentation procedure. Therefore, there is a need for the development of a classification system that fosters easy communication between practitioners and provides a prospective on the difficulty of the management of any particular case. The ideal classification system has to be comprehensive, practical and simple and it should provide a clinical recommendation on the management and /or the prognosis of the case. The proposed system is based on the orientation of the maxillary septum as identified in the CTCB scans. The different classes were assigned based on the prevalence of different orientation of the maxillary septa as reported in the literature, meaning that Class I septa is more prevalent than Class II or III. We also incorporated a Class IV that included septa which have a combination of orientation. In addition to the prevalence, we also considered the difficulty of the surgical management based on the incidence of complications.

The majority of sinuses in this study with interfering septa contained Class I septa (Coronal Plane), or septa with a buccal-lingual orientation that divided the sinus cavity into anterior and posterior compartments. This is in agreement with what was reported previously in studies that evaluated cadaver specimens and CBCT images [13, 14, 23, 24]. Park, *et al.* [23] evaluated CBCT images of 400 sinuses and found that out of the 111 sinuses that had septa, 106 of them had septa oriented in a buccal-palatal direction (Coronal Plane), four sinuses had septa in a sagittal direction, and one sinus had a septum in a transverse direction. On the other hand, Rosano, *et al.* [17] in a cadaver study evaluating 60 sinuses found that 70% of maxillary septa were in a sagittal direction.

A systematic review by Pommer, *et al.* [27] evaluated the morphology of maxillary sinus and in 87% of the cases, the septa had a buccal-palatal orientation. However, sagittal orientation was found in 11.1%, and horizontal septa were observed in 1.3% of cases. In this study, the incidence of Class II septa (sagittal) was 5.1%, and Class III (horizontal shelve) was 3.1%. Interestingly, we found that 5.1% of the cases had a combination of the different orientations of the septa and, therefore, were classified as Class IV. A similar finding has been reported by Sakhadri, *et al.* who found a small number of their subjects (1%) to have 2 or three septa directed in different orientations [28]. The surgical manipulation of Class V has been difficult and resulted in membrane perforation in all of the cases. To our knowledge, the only proposed classification for maxillary septa was by Wen, *et al.* [9] and it was based on the degree of difficulty of the surgical manipulation of the case. Cases were categorized into easy, moderate or difficult class and each of the cases were sub-classified into A, B and C based on factors like the location, number, orientation and size of the septa. A proposed clinical guideline into how to manage different cases was given. However, in this classification, only septa that have a mediolateral or antero-posterior direction were included, and septa oriented in other directions were not considered. Although the incidence of Class III and Class IV septa is relatively small, those cases are considered a real challenge to the implant surgeon and require an advanced level of skills and experiences to manage them.

The incidence of postoperative complications in this study was low and was not associated with membrane perforation. The incidence of reported infection following sinus lift surgery has ranged from 0 to 12.5% [25, 29, 30]. There has been conflicting information regarding the association between membrane perforation and the development of sinusitis. Nolan, *et al.* [7] found that 85% of sinuses developing sinusitis and secondary infection had intra-surgical membrane perforation. Others had argued that the occurrence of sinusitis is not associated with the occurrence of membrane perforations [31, 32]. Four types of bone grafts material were available and the selection of which graft is used during the procedure was based on the clinical judgment of the operator. Injectable paste material has the advantage of reducing operating time with a clinically acceptable result [33]. However, in this study, it was associated with a higher incidence of postoperative complication rate. This has to be interpreted with caution due to the limited number of sinuses and the retrospective nature of the study. In the literature, an association between the type of bone graft material used during sinus lift surgery and the occurrence of postoperative complications has not been found [34]. Postoperative pain in the sinus and infraorbital area is common and could last up to 3 weeks [35]. Soft tissue dehiscence results from poor flap management and may result in graft loss [35].

The occurrence of a sinus membrane perforation was related to implant marginal bone loss at the latest follow-up. However, this association was not found to be statistically significant. Karabuda, *et al.* [36] assessed the effect of sinus membrane perforation on the success of implants placed in augmented sinuses in 91 patients using 259 implants. Both one and two stage direct sinus lifts were included. A statistically significant difference was not detected in the peri-implant resorption rate between implants placed in sinuses with or without membrane perforations [36]. Although some evidence in the literature points to a relationship between sinus membrane perforations and reduced implant survival [5, 18] the majority of research on this topic points to the lack of such an association [2, 6]. The trend of sinus membrane perforations causing increased marginal bone loss following implant placement, even if clinically relevant, may never prove to be statistically significant because the actual increase in marginal bone loss caused by a perforation is likely to be very small relative to the overall success of implant therapy in the posterior maxilla.

Insufficient bone level is a frequent problem encountered during the restoration of posterior maxilla. Several sinus augmentation techniques *i.e.* lateral window and crestal approach, the use of autogenous bone graft or bone substitute or the use of short dental implant have been proposed. To date, there is no conclusive evidence in the literature on the superiority of one technique over the others in terms of prosthetic or implant success [37]. Short dental implants or crestal approach might offer an alternative to lateral window approach when residual bone height is 4-9 mm with lower complications rate [37]. However, when advanced resorption of alveolar ridge is encountered, lateral window sinus augmentation is a reliable and well documented technique providing that careful evaluation of the risk factors and technical details are considered. This study has several limitations being a retrospective review of a limited number of sinus augmentation procedures. Although all procedures were carried out by one clinician, different types of bone grafts were used. Bone loss was measured from radiographs taken at implant placement and at follow up but due to the relatively short follow-up period following integration check, the detection of significant differences or correlations between contributing factors and marginal bone loss was made difficult. In addition, the restoration of dental implants plays a significant role in their overall success. Factors such as the creation of hygienic implant restoration contours as well as proper occlusal adjustment are critical to their long-term success. The majority of patients in this study returned to their referring dentists for implant restoration, and thus the confounding variable of multiple operators with different skill levels may also have affected the results observed.

## CONCLUSION

Sinus septa should not be considered as a contraindication to sinus lift surgery. Septa should be identified, classified and managed with a meticulous attention to technical details. A classification based on the septal orientation is proposed since the orientation of the septa can complicate the surgical procedure and requires modification of the surgical technique. In this study, the identification of interfering septa on preoperative CBCT scans was significantly associated with the occurrence of intra-surgical sinus membrane perforations. However, sinus membrane perforations were not significantly associated with major post-operative complications or implant marginal bone loss.

## Figures and Tables

**Table 1:** Patients demographic, smoking and medical history and type of procedure as its relate to the incidence of membrane perforation and postoperative complications.

	Number (%)	Incidence of Membrane Perforation (%)	Fisher’s Exact Test	Incidence of Other Complications (%)	Fisher’s Exact Test
**Age**					
<45	12 (18%)	3 (25%)		1 (8.3%)	*P*=1.000
45-64	37 (55%)	8 (21%)		5 (13.5)	
>65	18 (27%)	7 (38.9)	*P*=0.422	2 (11.1%)	
**Total**	**67**				
**Gender**					
Male	25 (37.3%)	7 (28.0%)	*P*=0.700	2 (8.0%)	
Female	42 (62.7%)	11 (26.2%)		6 (14.3%)	*P*=0.700
**Smoking history**					
Non smoker	59 (88%)	15 (25.4%)	*P*=0.672	8 (13.6%)	*P*=0.582
Smoker	8 (11.9%)	3(37.5%)		0 (0%)	
**Medical history**					
Non-significant	48 (71.6%)	12 (25%)	*P*=0.760	7 (14.6%)	*P*=0.424
>= 1 medical condition	19 (23.35%)	6 (31.6%)		1 (5.3%)	
**Procedure**					
Sinus lift alone	48 (60.75%)	9 (18.8%)	*P*=0.410	5 (10.4%)	*P*=1.000
Sinus lift + implant	31 (39.24%)	9 (29.0%)		3 (9.7%)	
**Total**	**79**	18 (22.78%)		8 (10%)	

**Table 2:** The occurrence of membrane perforation as its related to the identification of interfering septa in CBCT, and the incidence of membrane perforations, according to septal class once an interfering septum was identified in CBCT. The presence of septum was significantly associated with intra-surgical membrane perforation. *Fisher’s Exact Test *P* < 0.001.

	Membrane Perforation		**TOTAL**
	No Perforation	Perforation	
**No septa identified**	40 (97.6%)	1 (2.4%)	41
**One or more septa**	22(55.3%)	17 (44.7)*	38
**TOTAL**	62 (78%)	18 (22%)	79
**Septal classification**	No perforation	Perforation	TOTAL
Class I (medio-lateral)	16 (59.3%)	11 (40.7%)	27
Class II (antro-post)	2 (50.0%)	2 (50.0%)	4
Class III (Shelf)	3 (100.0%)	0 (0.0%)	3
Class IV (Combination)	0 (0.0%)	4 (100.0%)	4
**TOTAL**	21 (55%)	17 (44.7%)	38

**Table 3:** Proposed classification of maxillary sinus septa and clinical recommendation on management.

**Septal Classification**	**Orientation**	**Clinical Remarks**	**Surgeon Degree of Experience Needed**
**Class I**	Medial-lateral (Coronal Plane)	The most common orientation Careful surgical manipulation Difficulty of the case depends on the size and number of septa	Early -Moderate experience
**Class II**	Anterior-posterior (Sagittal Plane)	Second most common orientation Moderate difficultly based on location Higher incidence of membrane perforation	Moderate experience
**Class III**	Horizontal or shelf-like (Transverse Plane) off one of the medial or lateral walls	Least common orientation Difficultly based on location /size Higher incidence of membrane perforation	Significant experience
**Class IV**	A combination of Class I, II, or III.	Common incidence Difficult management with high incidence of membrane perforation. Requires modification of the surgical technique and /or special instrument development	Significant experience /advanced technology

**Table 4:** Overall averaged marginal bone loss and bone loss at the 1.5 and 1.0 mm thresholds for sinus membrane perforations. * Fisher’s Exact Test *P*=0.410. ** Fisher’s Exact Test *P*=0.215.

	**No Membrane Perforation**	**Membrane Perforation**
Bone loss <1.5 mm* Number (%)	25 (89.3%)	6 (66.7%)
**Bone loss ≥1.5 mm** Number (%)	3 (10.7%)	3 (33.3%)
Bone loss <1.0 mm** Number (%)	22 (78.6%)	5 (55.6%)
**Bone loss ≥1.0 mm** Number (%)	6 (21.4%)	4 (44.4%)

## ABBREVIATION

CBCT: = Cone Beam Computed Tomography
